# Development and assessment of an intestinal tri-cellular model to investigate the pro/anti-inflammatory potential of digested foods

**DOI:** 10.3389/fimmu.2025.1545261

**Published:** 2025-02-05

**Authors:** Marina Ramal-Sanchez, Chiara Bravo-Trippetta, Veronica D’Antonio, Elena Corvaglia, Angela A. M. Kämpfer, Roel P. F. Schins, Mauro Serafini, Donato Angelino

**Affiliations:** ^1^ Functional Foods and Stress Prevention Laboratory, Department of Biosciences and Technology for Food, Agriculture and Environment, University of Teramo, Teramo, Italy; ^2^ IUF – Leibniz Research Institute for Environmental Medicine, Düsseldorf, Germany

**Keywords:** immunonutrition, cytokines, nutrition, gut, IBD, tri-culture, advanced cellular model, inflammation

## Abstract

**Introduction:**

Immunonutrition, defined as the potential of foods, nutrients and dietary patterns to modulate the immune system activity, has been proposed as a strategy to enhance the immune response in both metabolic and immune-mediated diseases. However, the anti-/pro-inflammatory role of foods and diets is far to be fully ascertained, and thus there is a continued needed for appropriate in vitro cell-culture models to investigate the role of foods in modulating cell-mediated inflammatory processes. This study aims to develop and test an in vitro tri-culture model, simulating the complexity of the intestinal tract and its multiple cell interactions.

**Methods:**

To achieve this, the intestinal epithelial barrier was established by co-culturing human Caco-2 enterocyte-like and HT29-MTX-E12 mucus producing goblet-like colon cells, then adding human monocyte THP-1 cells to the basolateral compartment. The integrity and stability of the epithelial barrier were monitored and the inflammatory response of the model was assessed using various stressors at different concentrations, both individually and in combination (phorbol-12- myristate-13-acetate or PMA, and lipopolysaccharide or LPS), in terms of cytokines production. To test the model, different concentrations of *in vitro* digested broccoli (BD) were added to the apical section of the model.

**Results:**

Supernatants from the basolateral compartment were collected and analyzed for cytokines production (IL-6, TNF-α, IL-12p70, IL-18 and IL-8) using automated ELISA (ELLA). Additionally, ZO-1 protein from the tight junctions of epithelial cells was analyzed by flow cytometry. The results indicated that 100 nM PMA added to the whole model for 20 h was the best stressor to simulate a mild-inflammatory status of the gut. Following treatment with BD, IL-6, TNF-α, IL-8 and IL-18 were significantly reduced compared to the control group, while ZO-1 expression increased at the lowest BD concentration.

**Conclusions:**

These findings confirm the feasibility of the model for assessing the effects of food digesta on specific cytokines and permeability markers, representing a valuable strategy for investigating the role of foods in modulating the inflammatory response. The results obtained may support dietary strategies aimed at promoting wellbeing and preventing inflammatory-related metabolic diseases.

## 1 Introduction

Over the past few decades, lifestyle factors as smoking/drinking habits, physical inactivity and the adherence to unbalanced dietary patterns – characterized by a high intake of foods rich in (saturated) fats, sugars, salt and a low intake of foods source of fiber and plant-based proteins – has led to an increased incidence of chronic metabolic diseases such as obesity, diabetes and cardiovascular diseases (CVDs), and relative deaths (1, 2). These pathologies are all characterized by a common disruption of the immune system, known as meta-inflammation, defined as a “chronic, low-grade systemic inflammation and differing from the acute inflammatory response typical of the innate immune system” (3).

Among the chronic inflammatory intestinal disorders, Crohn’s disease (CD) and ulcerative colitis (UC) are the major representatives of inflammatory bowel diseases (IBDs) (4). While the causes are still unknown, a combination of genetic, immunological and environmental factors may be the triggers of the excessive and abnormal immune response, leading to a wide variety of symptoms and a low quality of life for the patient (5). The initiation of the immune response in CD and UC patients is characterized by an increased migration of monocytes into areas of inflammation, which differentiate then into a pro-inflammatory macrophage phenotype (M1), releasing pro-inflammatory cytokines that amplify the immune response and attract additional cellular and humoral components of the immune system (6, 7). Notably, a strong correlation has been demonstrated between obesity and IBDs (8, 9). While low-grade inflammation is present in obese individuals even without other pathological conditions, a persistent metabolic inflammation has been associated with the development of comorbidities, such as IBDs, type 2 diabetes (T2D) or CVDs (10, 11).

Diet plays an important role in modulating cytokines-induced stress associated with the raising of inflammatory chronic conditions. Specific macronutrients from the unbalanced dietary patterns have been proposed as fuel for the inflammatory response in the gut, perturbing not only the innate immunity but also the gut microbial profile and metabolism (4). In contrast, dietary patterns characterized with a high intake of fruit, vegetables, legumes, whole grain products, and low consumption of animal-based products has been associated with lower levels of chronic inflammation (12–15). Scientific research focused on human studies has shown a protective role for a high intake of plant foods and inflammatory markers of inflammation, as C-reactive protein, interleukin 6 (IL-6) and tumor necrosis factor alpha (TNF-α) (13).

In order to unravel the potential of various food items in modulating the inflammatory response, several *in vitro* gut-mimicking models have been proposed. To date, literature suggests that the most frequently used model is represented by a single-cell culture, mainly represented by the enterocyte or colonocyte (16, 17). However, it represents a simplistic strategy, restricted to the study of preliminary tests on specific situations and well-known mechanisms, omitting the interaction with immune cells and their key participation in inflammatory and immune processes (18). In an effort to solve this issue, a two-cells approach including intestinal and immune cells has been proposed as a valuable alternative (19–22). Nevertheless, the intestinal tract is a complex environment characterized by many interactions among different cells with specific features, underlining the need for using an *in vitro* model able to resemble the human intestine and the interactions between the main participating cells as much as possible, but simple enough to exclude the variability related to the single individual characteristics.

Recently, a more complex culture model composed of three cell lines (tri-culture model or 3C henceforth) has been developed to *in vitro* simulate the physiological inflammatory processes occurring *in vivo* at gut level, within limits (23–26). This tri-culture model is composed by epithelial cells from i) Cao-2, an immortalized human colon carcinoma cell line; ii) HT29-MTX-E12, a human colon intestinal cell clone able to produce mucus; and iii) the monocyte cell line THP-1, isolated from the peripheral blood of a patient affected by acute monocytic leukemia and widely used in immunology research. 3C model can be assembled by co-culturing epithelial cells both in the apical part of the insert and THP-1 in the basolateral compartment, without physical interaction among the cells, or in an inverted position, by culturing epithelial cells in the basolateral surface of the insert and the THP-1 cells in the apical part, allowing a direct interaction among the cell lines. 3C has been already used for different research purposes, as for the screening and evaluation of therapeutic drugs for IBD treatment (23), or to screen potential anti-inflammatory compounds in the context of intestinal inflammation, inducing a mild inflammatory status after lipopolysaccharide (LPS) addition (25). Kämpfer and collaborators compared the potential toxicity of different engineered nanomaterials (ENMs) on monocultures of Caco-2 and HT29-MTX-E12 cells and on the 3C model including THP-1 cells, confirming that 3C model represent a more adequate strategy to address advanced and multifaceted research questions (24). The same model but in an inverted orientation was used by Busch and colleagues to elucidate intestinal effects of low-density polymeric particles from environmental pollution, finding effects that previously were absent in the non-inverted model (27).

Last but not least, the majority of the approaches have tested foods just with water/organic solvent extraction processes, without undergoing a process of digestion, as happens in “real-life” conditions (28–30). This approach might have led to unreliable results due to the biochemical and/or metabolic transformation that occur during cooking and/or digestion. To overcome this problem, in the development of our model the tested food extracts were subjected to *in vitro* digestion.

The aim of this study is to develop and optimize a tri-culture *in vitro* cellular model, including intestinal and immune cells and simulating a mild-inflammatory status, to assess the pro/anti-inflammatory role of digested foods. To accomplish the aim, the model setting-up has been subdivided into different steps: a) establishment and assessment of the intestinal epithelial barrier composed by Caco-2 and HT29-MTX-E12 cells; b) selection of the baseline inflammatory conditions in the immune component of the model (THP-1 cells); c) tri-culture model assembly and evaluation; d) selection of the baseline inflammatory conditions in the tri-culture model, including an anti-inflammatory drug and the final cytokine pattern; e) analysis of the anti/pro-inflammatory response testing a food digesta on the inflamed tri-culture model; f) quantification of cell lysis by measuring LDH release; and g) evaluation of ZO-1 protein expression on the intestinal epithelial barrier.

## 2 Materials and methods

### 2.1 Chemicals

Unless otherwise stated, chemicals were acquired from Sigma-Aldrich (Saint Louis, MO, USA).

### 2.2 Intestinal epithelial cells co-culture and maintaining

Caco-2 cells (cat. no. 09042001-1VL, ECACC) and HT29-MTX-E12 cells (ECACC, 12040401) were cultured in DMEM cell culture medium supplemented with 10% FBS, 1% penicillin/streptomycin and 1% non-essential amino acids (NEAAs), maintained at 37 ˚C, 5% CO_2_, humified atmosphere. Caco-2 and HT29-MTX-E12 cells were regularly split at ~80% confluence and used at passages 10–30 after thawing for co-culture experiments.

For intestinal epithelial cells co-culture, cells were seeded on Transwell inserts (1 μm pore size; Falcon, Sacco S.r.L., Cadorago, Como, Italy) at a density of 1.66*10^5^ cells per well in a ratio 9:1 (Caco-2:HT29-MTX-E12) and maintained up to 21 days, renewing the medium on Monday, Wednesday and Friday. On the apical (AP) side, cells were cultured in DMEM, whereas the medium in the basolateral (BL) compartment was progressively changed to RPMI-based THP-1 medium without β-mercaptoethanol.

### 2.3 Trans-epithelial electrical resistance monitoring

To monitor the intestinal barrier integrity and stability, trans-epithelial electrical resistance (TEER) measurements were performed at 7, 14 and 21 days after the beginning of the co-culture using the volt-ohm meter Millicell^®^ ERS-2 (Electrical Resistance System, Merck Millipore). The resistance has been then calculated and expressed as Ohm per cm^2^ filter surface, following the equation:


$$\text{Resivity}(\text{Ω}\;\text{c}{\text{m}}^{2})=(\text{Ohm}2-\text{Ohm}1{)}^{*}\text{A}$$

where Ohm1 is the blank resistance, Ohm2 the insert resistance and A the surface area of the insert.

### 2.4 Immune cells culture and stimulation

THP-1 (ATCC, TIB-202) cells were cultured in RPMI 1640-based cell culture medium (containing L-glutamine and 25 mM HEPES) supplemented with 10% FBS, 1% penicillin/streptomycin, 1 nM sodium pyruvate, 0.7% d-glucose and 50 nM β-mercaptoethanol and maintained between 2*10^5^ and 8*10^5^ cells/mL, at 37 ˚C and 5% CO_2_, humified atmosphere.

To stimulate THP-1 differentiation into a pro-inflammatory macrophage phenotype, different concentrations of well-known stressors have been used, in multiple combinations. To that, THP-1 cells have been cultured in 12-well plates (2*10^5^ cells per well) during 24 hours under culture conditions, adding lipopolysaccharide (LPS) at 100 ng/mL and/or phorbol 12-myristate 13-acetate (PMA) at 50, 75 and 100 nM, added in contemporary (24 hours of inflammation) or consecutively (24h LPS + 24h PMA, reaching a total of 48 h of inflammation). The inflammatory response of the different study groups has been measured in terms of TNF-α and IL-6 production.

### 2.5 Tri-culture model assembling and stimulation

**Figure 1:**
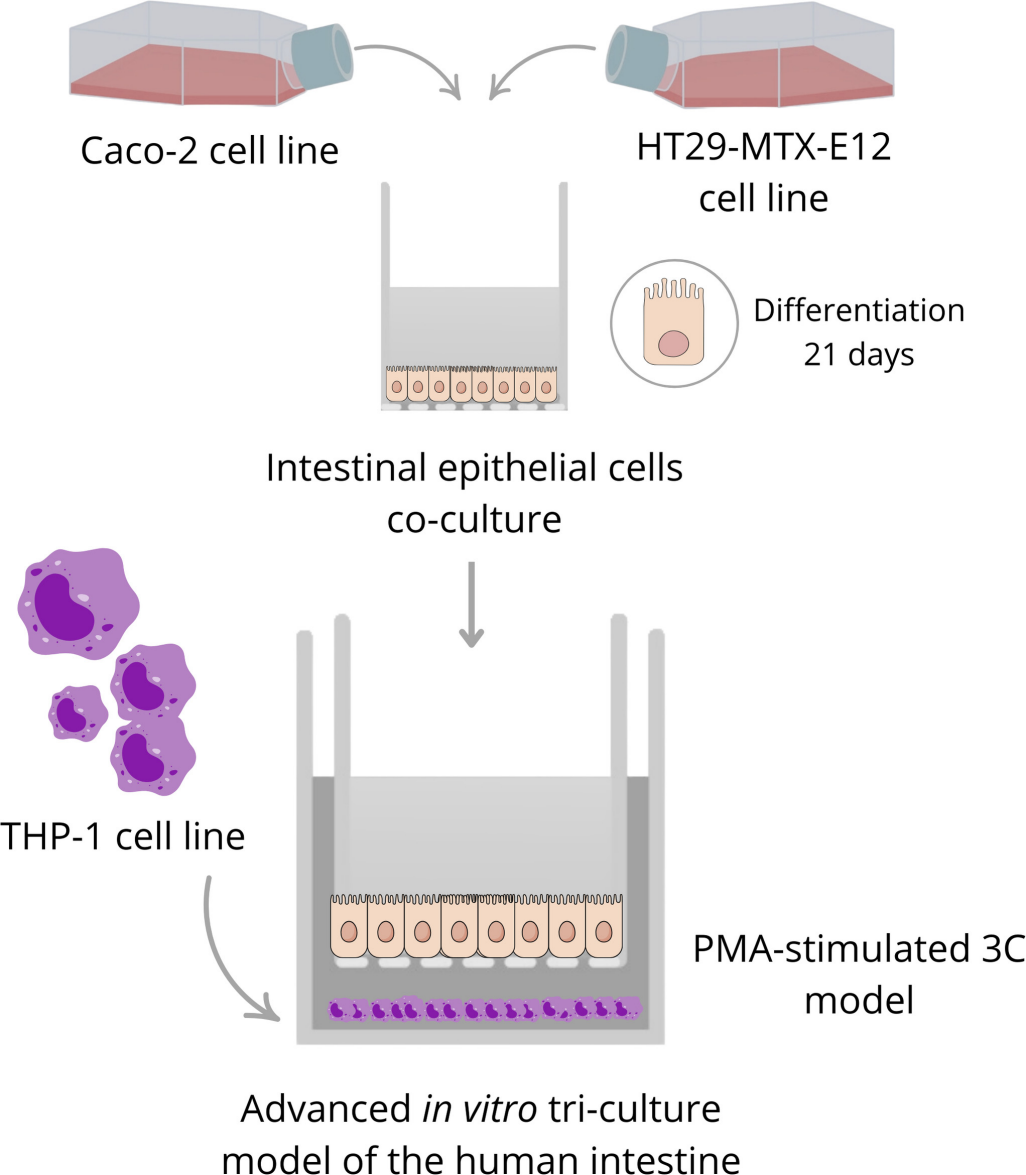
Schema of the tri-culture model assembling.

Tri-culture model has been assembled by transferring the Transwell inserts onto the THP-1 cells, immediately after seeding them in 12-wells plates at a density range of 2*10^5^ cells per well with the different treatments: no treatment (non-differentiated THP-1 cells); LPS (100 ng/mL); PMA (100 nM); PMA + LPS (at the same concentrations and in contemporary). The inflammatory response of the different study groups has been measured in terms of TNF-α and IL-6 production. To decipher the best anti-inflammatory drug concentration, a dose-concentration analysis of the glucocorticoid prednisolone was performed (5, 1, 0.5 and 0.25 µM). To explore the potential role of the enzymes present in the food digesta (see section 2.6), a digesta with no food was examined as well, at different concentrations (500, 400, 200, 100 and 50 µL per mL of supplemented-DMEM). A schema of the tri-culture model assembling is available in **Figure 1**.

### 2.6 Preparation of food digesta and calculation of dry matter concentration

Broccoli digesta (BD) were used as sampling digesta to set the tri-culture model. Specifically, three different cultivars/varieties of the product purchased in local supermarkets/groceries have been washed, steamed for 5 min, then dabbed with paper towel; after mincing, the pool from the different cultivars were considered as unique sample for *in vitro* digestion. Dry matter of samples was determined according to the AOAC gravimetrical method (31).

Digestion protocol referred to the harmonized INFOGEST static *in vitro* digestion procedure, simulating the physiological conditions of the oral, gastric and small intestinal digestion phases *in vitro* (32), with opportune modifications.

The oral phase was carried out by using human saliva collected from healthy volunteers, according to Chen et al. (33). The fresh saliva samples were collected after 2 hours from the last meal. The donors were invited to rinse their mouth with deionized water for at least 30 sec to obtain a neutral environment and then saliva at the first 30 s was discarded. Saliva was collected in the next 5 min each 30 sec, until the needed amount was reached. The collected saliva was immediately centrifuged at 1780 *x g* for 10 min and the supernatant used for the following procedure.

In order to simulate mastication, 4 mL of human saliva was added to 4 g of food sample and then the mixture was grinded with mortar and pestle for 2 min. The gastric phase was started by adding simulated gastric fluid containing 2000 U/mL pepsin in the final volume. The pH was adjusted to 3 and the volume to 16 mL and the mixture was incubated at 37°C for 2 h in a rotating mixer. Then, a solution containing simulated intestinal fluid, containing bile extract (10 mM of bile salts in the final volume) and pancreatin (100 U/mL of trypsin activity in the final volume) was added. The pH was adjusted to 7 and the volume to 32 mL and the mixture was incubated at 37°C for 2 h in a rotating mixer. Digesta were collected, centrifuged at 1780 *x g* and the supernatant was aliquoted and stored at -20°C until cell treatments. For testing, BD were diluted in supplemented-DMEM at different concentrations (400, 200, 100, 50 µL per mL of supplemented-DMEM). A digesta solution with no food (NF), but containing the enzymes and subjected to the standard procedure described, was used as a blank for the experiments evaluating the digesta effects.

### 2.7 Anti-inflammatory drug selection and digesta enzymes evaluation

To decipher the best anti-inflammatory drug concentration to be used, a dose-concentration analysis using a glucocorticoid was performed. Different concentrations of prednisolone were used: 5, 1, 0.5 and 0.25 µM. In addition, the NF solution at different concentrations (500, 400, 200, 100, 50 µL per mL of supplemented-DMEM) was tested as well in terms of cytokines production. After the stimulation of the model with PMA at 100 nM for 20 hours, treatments were added to the apical part of the model and incubated for 4 hours under culture conditions. Then, the supernatants from the basolateral compartments were recovered and stored at -80°C until the analysis.

### 2.8 Cytokines analysis by traditional ELISA or ELLA Simple Plex^®^

To select the final cytokine pattern to be used, the whole 3C model was stimulated with 100 nM PMA during 24 hours (PMA-CTRL) and the supernatants from the basolateral compartment were compared with those from a non-stimulated 3C model (CTRL). Prednisolone and NF digesta were used to evaluate potential cytokines differences, tested on the model as previously described. The supernatants from the basolateral compartments were recovered and stored at -80°C until the analysis.

For the model setting-up and the selection of the cytokine pattern, TNF-α, IL-6, IL-1β, IFN-γ, IL-12p70, IL-23, CCL20, IL-18 and IL-8 cytokines were analyzed using traditional Human Quantikine ELISA kits (Bio-Techne, Minneapolis, MN, USA), following the manufacturer instructions and measuring with the spectrophotometer (Enspire, Perkin Elmer). Once the pattern was identified, for digesta testing, TNF-α, IL-6, IL-12p70, IL-18 and IL-8 cytokines were analyzed using ELLA Simple Plex**^®^** automated immunoassay system (ProteinSimple, San Jose, CA, USA), using commercially available customized simple plex kits and according to the manufacturer instructions.

### 2.9 Evaluation of cell lysis by LDH quantification assay

Cell damage (or necrotic cell death) has been measured by quantifying the enzymatic activity of the lactate dehydrogenase (LDH) released. Briefly, 50 μL of 200 mM TRIS, 50 μL of 50 mM lithium lactate, and 50 μL mix of iodonitrotetrazolium (INT), phenazine methosulfate (PMS), and nicotinamide adenine dinucleotide (NAD) at 1.32 mg/mL, 0.36 mg/mL and 3.44 mg/mL concentrations, respectively, were added to a 96-wells plate. Subsequently, 50 μL of cell-free supernatants have been added to the previous mix and incubated for 5 min at room temperature (RT). As a control of 100% cell lysis, cells exposed to 0.1% Triton X-100 in PBS for 24 h have been used. Optical density has been spectrophotometrically measured (Enspire, Perkin Elmer) at 490 nm. A background control in complete cell culture medium (CCM) was subtracted from the results.

### 2.10 Analysis of ZO-1 protein expression

To analyze ZO-1 protein expression on the intestinal epithelial barrier, cells were recovered from the inserts using Accutase**^®^**, then washed and fixed using a 4% paraformaldehyde (PFA) solution. For flow cytometry analysis, fixed cells were incubated with an anti-ZO-1 antibody produced in rabbit for 1 h, and then with a goat anti-rabbit IgG Alexa Fluo 488 (ThermoFisher Scientific) for 1h. Samples were then analyzed using a CytoFLEX flow cytometer with 488 nm and 638 nm wavelength lasers (B53013) and operated with the CytExpert software (Beckman Coulter, Brea, CA, USA). Events were acquired exciting with the 488 nm laser light and using the band-passes of 525/40 nm for Alexa Fluor 488. Positive events were considered those expressing the protein ZO-1. Flow rate was adjusted to 100 events/sec, acquiring at least 5000 events for each independent sample. Data were extracted and analyzed with CytExpert, considering the fluorescence median from each sample group and comparing every treatment group to the control group. Experiments were performed in biological triplicate.

### 2.11 Statistical analysis

Statistical analysis was performed on data from at least 3 independent biological and technical experiments. Data were obtained using Graphpad Prism 9.1.0 and expressed as violin or histogram plots. To evaluate the treatment effect, delta value of cytokines was calculated by subtracting the control value to every treatment value. Normality tests (Shapiro-Wilk test for n<50) and outlier identification (ROUT) were carried out for every individual experiment. For cytokines analysis, ANOVA with Dunnett’s multiple comparisons or Friedman tests were performed; for the cytokines analysis following BD treatments, one-way ANOVA with Geisser-Greenhouse correction and uncorrected Fisher’s LSD was applied (information available for every figure legend).

## 3 Results

### 3.1 Establishment and assessment of intestinal epithelial barrier

**Figure 2:**
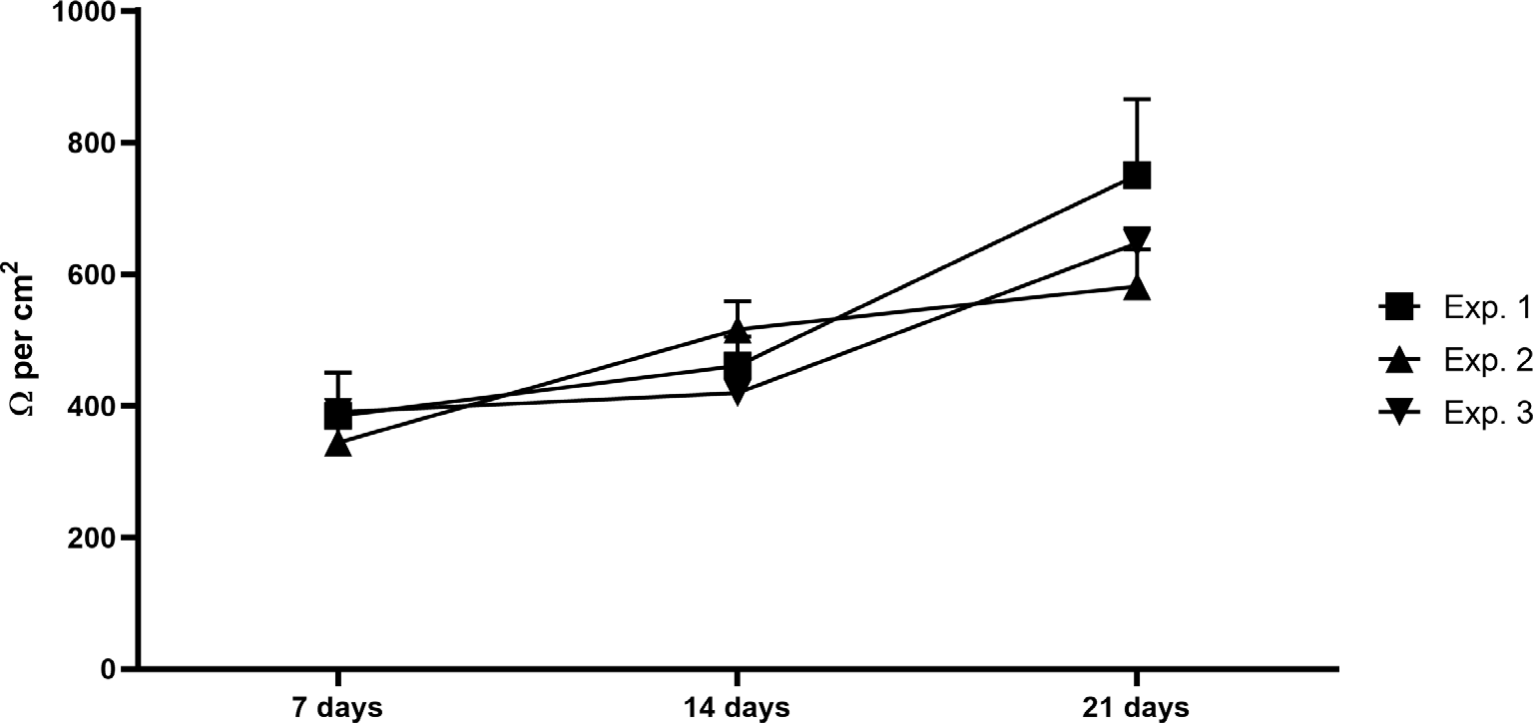
Intestinal barrier integrity. Graph shows the TEER values of the intestinal epithelial barrier composed by Caco-2 and HT29-MTX-E12 after 7, 14 and 21 days, confirming the cells growing and differentiation and the barrier stability. Exp, experiment; N= 3 independent experiments; every experiment corresponds to the mean of at least 10 different wells.

Intestinal epithelial co-culture has been monitored up to 21 days after the beginning of the co-culture in terms of barrier integrity and stability, confirming the expected cell differentiation and barrier stability along the culture, reaching TEER values of 600-700 Ω per cm^2^ at the end of the co-culture (**Figure 2**).

### 3.2 Baseline inflammatory conditions in THP-1 cells

**Figure 3:**
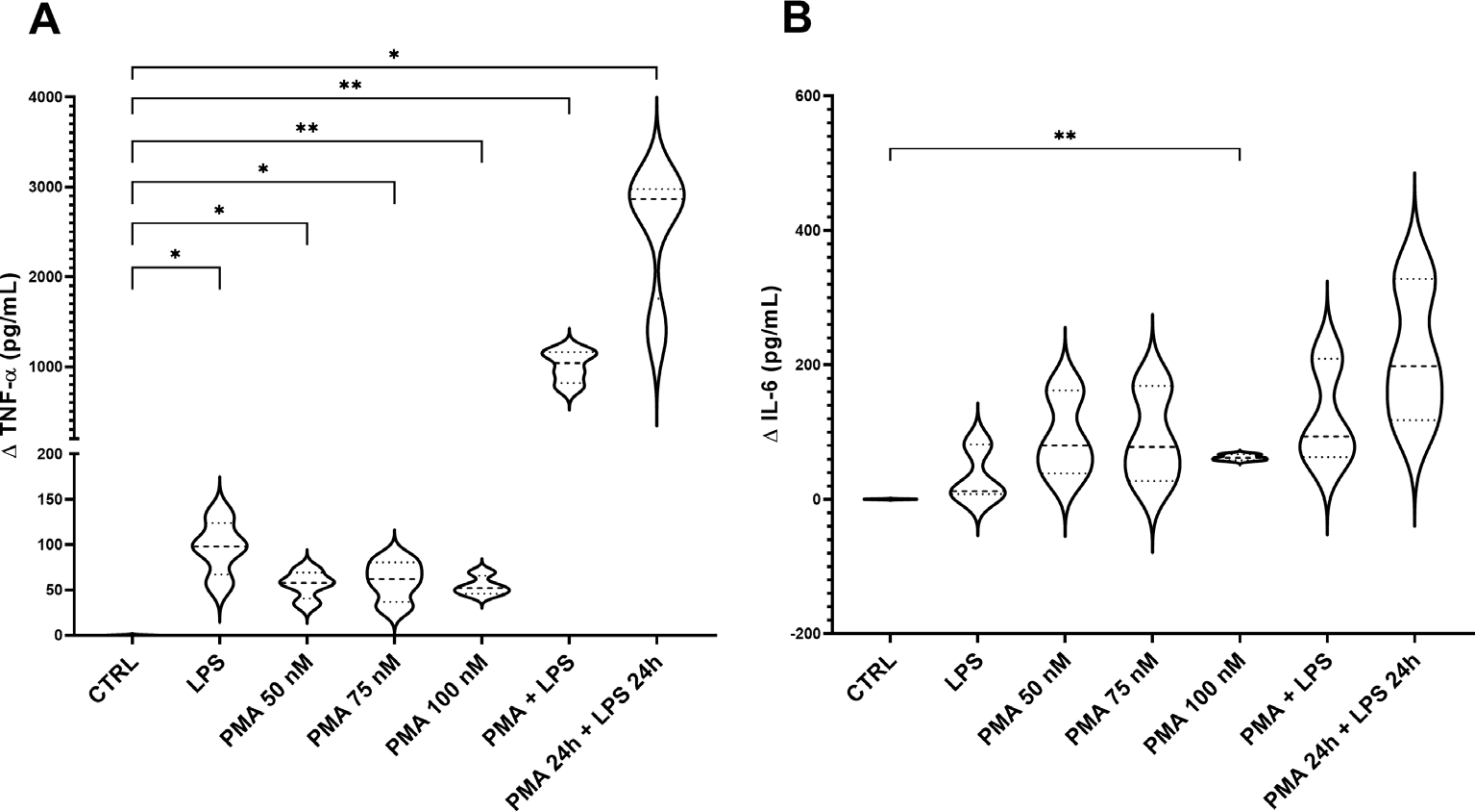
Concentration of TNF-α **(A)** and IL-6 **(B)** in the supernatant of stimulated-THP-1. The graph shows the delta values of TNF-α and IL-6 for each experimental group: THP-1 treated with LPS (100 ng/mL), PMA at different concentrations (50, 75, 100 nM), PMA + LPS (100 nM and 100 ng/mL, respectively) and PMA + LPS at the same concentrations but in a consecutive period (24 h + 24 h). CTRL: non-stimulated THP-1; PMA, phorbol-12-myristate-13-acetate; LPS, lipopolysaccharide. N=4. *p<0.05; **p<0.01, with respect to the CTRL group (one-way ANOVA, Dunnett’s multiple comparisons test).

Stimulated-THP-1 cells either with LPS (100 ng/mL), PMA (50, 75 or 100 nM) or 100 nM PMA in combination with LPS (100 ng/mL) produced concentrations of TNF-α significantly different compared to the control group, and particularly higher for 100 nM PMA and the combo PMA+LPS (results represented as delta value, **Figure 3A**). Regarding IL-6, significantly higher concentrations were observed for 100 nM PMA group compared to control (**Figure 3B**). In conclusion, 100 nM PMA was identified as the best option simulating a mild-inflammatory status.

### 3.3 Inflammatory conditions in the 3C model

**Figure 4:**
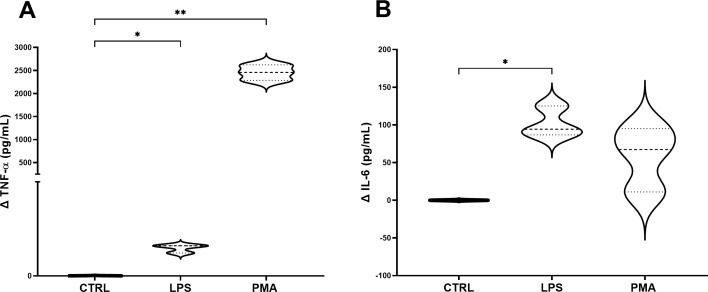
Concentration of TNF-α **(A)** and IL-6 **(B)** in the basolateral supernatant of the 3C model. Graphs show the delta values of TNF-α and IL-6 for each experimental group: non-stimulated 3C, 3C stimulated with LPS (100 ng/mL) and 3C stimulated with PMA (100 nM). CTRL: non-stimulated THP-1; PMA, phorbol-12-myristate-13-acetate; LPS, lipopolysaccharide. N=4. *p<0.05; **p<0.01, with respect to the CTRL group (one-way ANOVA, Dunnett’s multiple comparisons test).

To decipher the best stimulation conditions of the 3C model, once assembled the whole model was treated with PMA (100 nM) or LPS (100 ng/mL), added to the basolateral compartment. Results showed that there was a significant increase in concentrations following both stressor incubations in terms of TNF-α production, especially pronounced for PMA treatment (**Figure 4A**), compared to control. Regarding IL-6 concentrations, significant results were observed in the LPS group compared to control, showing for the PMA group a not significantly increase in IL-6 production than control, but it is worth reporting the higher variability in the concentration range (**Figure 4B**).

### 3.4 Dose-concentration analysis of prednisolone and digesta enzymes evaluation

**Figure 5:**
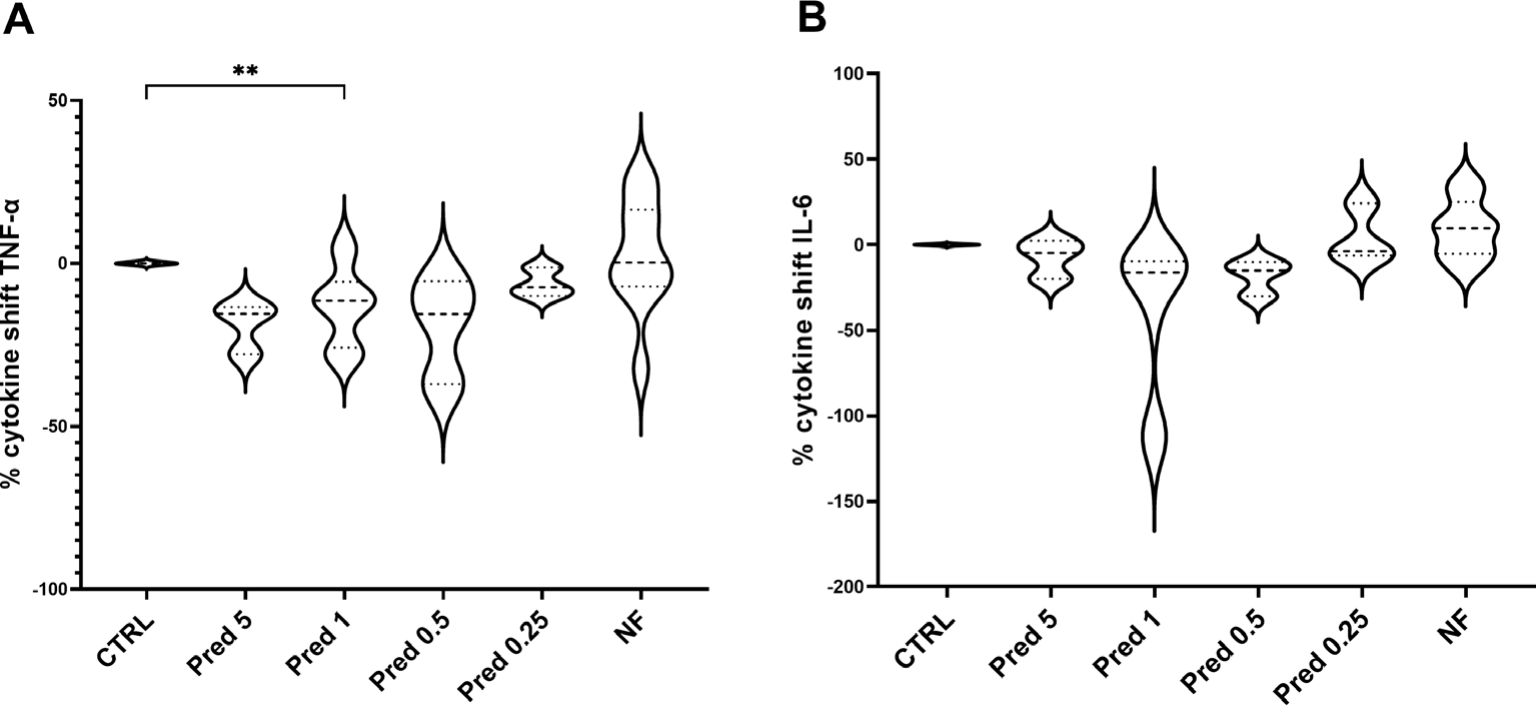
Dose-concentration of TNF-α **(A)** and IL-6 **(B)** following prednisolone and NF treatment. Graphs show the delta values of TNF-α and IL-6 for each experimental group: CTRL; prednisolone 5 µM, prednisolone 1 µM, prednisolone 0.5 µM, prednisolone 0.25 µM; digesta no food (NF) at 100 µL per mL. CTRL: PMA-stimulated THP-1; PMA, phorbol-12-myristate-13-acetate; N=3. **p<0.01, with respect to the CTRL group (one-way ANOVA, Dunnett’s multiple comparisons test).

Among the different studied concentrations of prednisolone (5, 1, 0.5 and 0.25 µM), 1 µM stand out as the best concentration to be used, showing significant results in the reduction of TNF-α compared to control and an absolute reduction of IL-6 compared to control, although not statistically significant. Moreover, values for digesta no-food showed no differences with respect to the control group. **Figure 5** shows the data obtained for NF at a specific concentration (100 µL per mL of supplemented-DMEM, as a representation of the concentration range studied (data not shown).

### 3.5 Selection and screening of the final cytokine pattern

**Figure 6:**
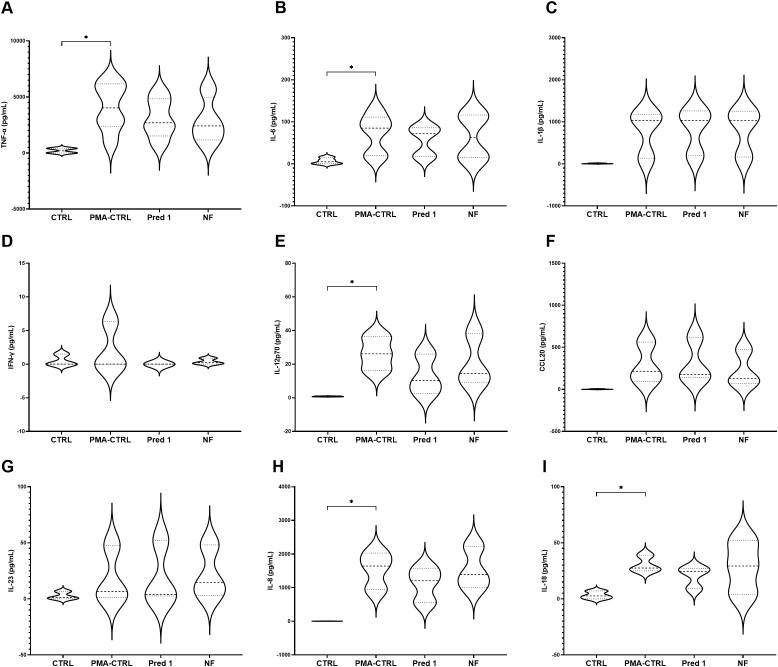
Cytokines production by the 3C model. Cytokines TNF-α **(A)**, IL-6 **(B)**, IL-1β **(C)**, IFN-γ **(D)**, IL-12p70 **(E)**, CCL20 **(F)**, IL-23 **(G)**, IL-8 **(H)**, IL-18 **(I)** production and comparison with ad without PMA stimulation. Graphs show the absolute concentration values for each experimental group: CTRL; prednisolone 5 µM, prednisolone 1 µM, prednisolone 0.5 µM, prednisolone 0.25 µM; digesta no food (NF) at 100 µL per mL. CTRL: non-stimulated THP-1; PMA-CTRL: 3C model stimulated with 100 nM PMA; Pred1: prednisolone 1 µM; NF, no-food digesta. PMA, phorbol-12-myristate-13-acetate. n=5 for TNF-α and IL-6; n=3 for the rest of the cytokines. *p<0.05, with respect to the CTRL group (one-way ANOVA, Dunnett’s multiple comparisons test).

Among the cytokines analyzed, TNF- α, IL-6, IL-12p70, IL-8 and IL-18 showed a significant increase when the model was stimulated with PMA at the selected concentrations, compared to the non-stimulated model. On the contrary, IL-1β, IFN-γ, CCL-20 and IL-23 showed no differences in terms of cytokines production (**Figure 6**).

### 3.6 Testing the 3C model in terms of cytokines production with a broccoli digesta

**Figure 7:**
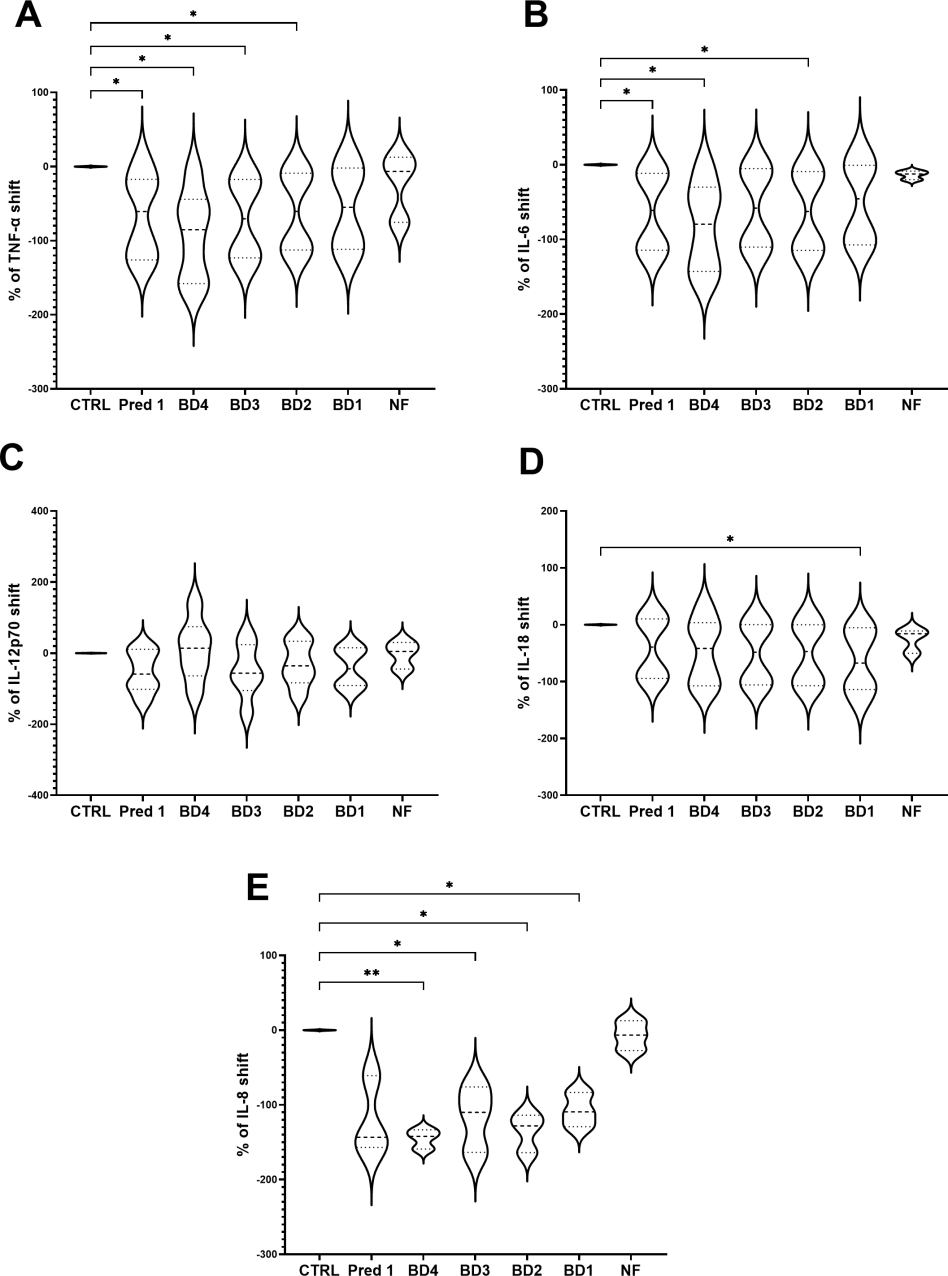
Cytokines shift following BD treatments. Graph shows the TNF-α **(A)**, IL-6 **(B)**, IL-12p70 **(C)**, IL-18 **(D)** and IL-8 **(E)** shifts following BD treatments to the PMA-stimulated 3C model. Results are expressed as cytokine shift with respect to the control group. Pred1: prednisolone 1µM; BD, broccoli digesta; NF, digesta no-food; PMA, phorbol-12-myristate-13-acetate. n=5. *p<0.05, **p<0.01, with respect to the control group (ANOVA with Geisser-Greenhouse correction and uncorrected Fisher’s LSD).

Broccoli digesta (dry-matter 9.67 g/L) were added to the 3C model and the production of TNF-α, IL-6, IL-12p70, IL-18 and IL-8 were assayed. Four concentrations of broccoli digesta were tested: BD4 (3.9 g/L), BD3 (1.9 g/L), BD2 (0.97 g/L) and BD1 (0.49 g/L). Significant differences were shown in TNF-α values with respect to the control group in all the BD concentrations, especially marked for BD4, BD3 and BD2 (**Figure 7A**), while IL-6 showed a significant reduction with BD4 and BD2 treatments compared to control (**Figure 7B**). Regarding IL-12p70, no significant differences were found for any food digesta (**Figure 7C**), while IL-18 showed a decrease with the lowest BD concentration (BD1), compared to the control group (**Figure 7D**). IL-8 showed a significant reduction with all the BD concentrations, particularly observed with the highest concentration (BD4) (**Figure 7E**).

### 3.7 Quantification of cell lysis after treatments by LDH assay

**Figure 8:**
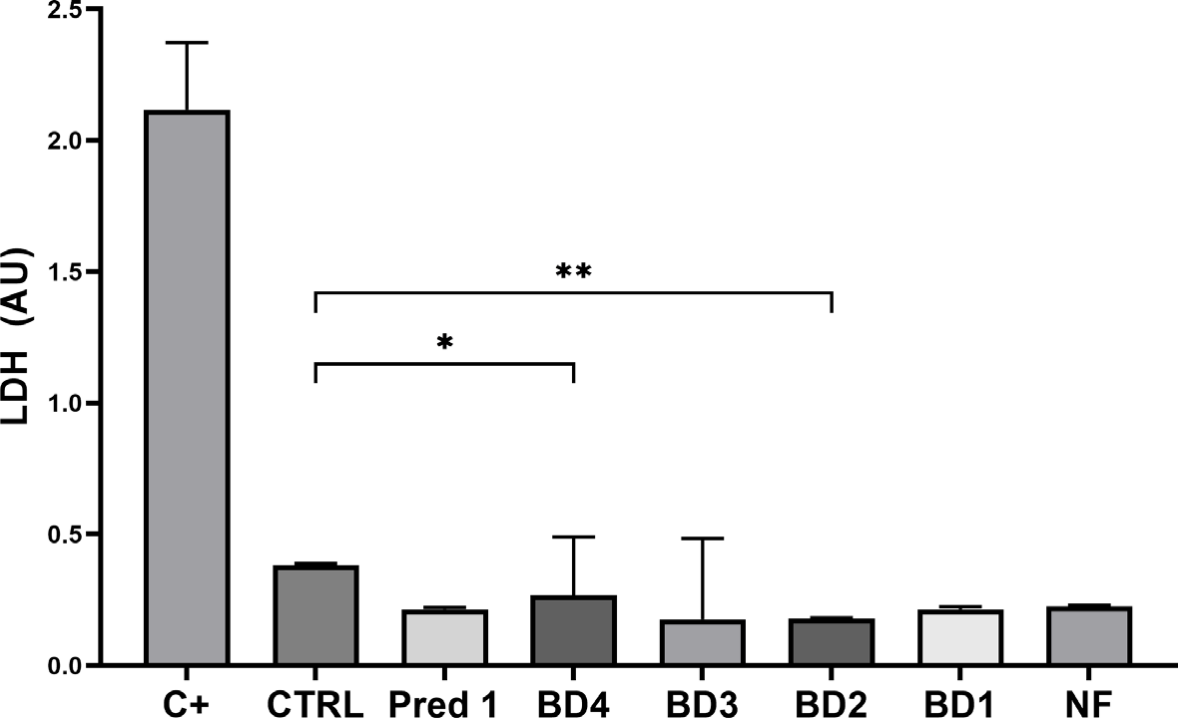
Quantification of cell lysis. Graph shows the concentration of LDH released by the intestinal epithelial cells from the apical compartment, compared to the control group. C+, positive control treated with 0.1% Triton x100; CTRL, control group; Pred1, prednisolone 1 µM; BD, broccoli digesta; NF, digesta no-food. LDH is expressed as arbitrary units (AU) following optical density measurement by spectrophotometry. Data are represented as Mean ± SD; n= 3 independent biological experiments; every independent experiment is the result of at least 4 technical replicates. *p<0.05; **p<0.01, with respect to the CTRL group (ANOVA, Friedman test).

Quantification of cell lysis of intestinal epithelial cells after BD treatments showed similar values to those from the control group (stimulated with PMA but not treated with BD), obtaining a significant reduction in LDH release on BD4 and BD2 groups compared to control (**Figure 8**).

### 3.8 Analysis of ZO-1 expression on intestinal epithelial cells following broccoli digesta

**Figure 9:**
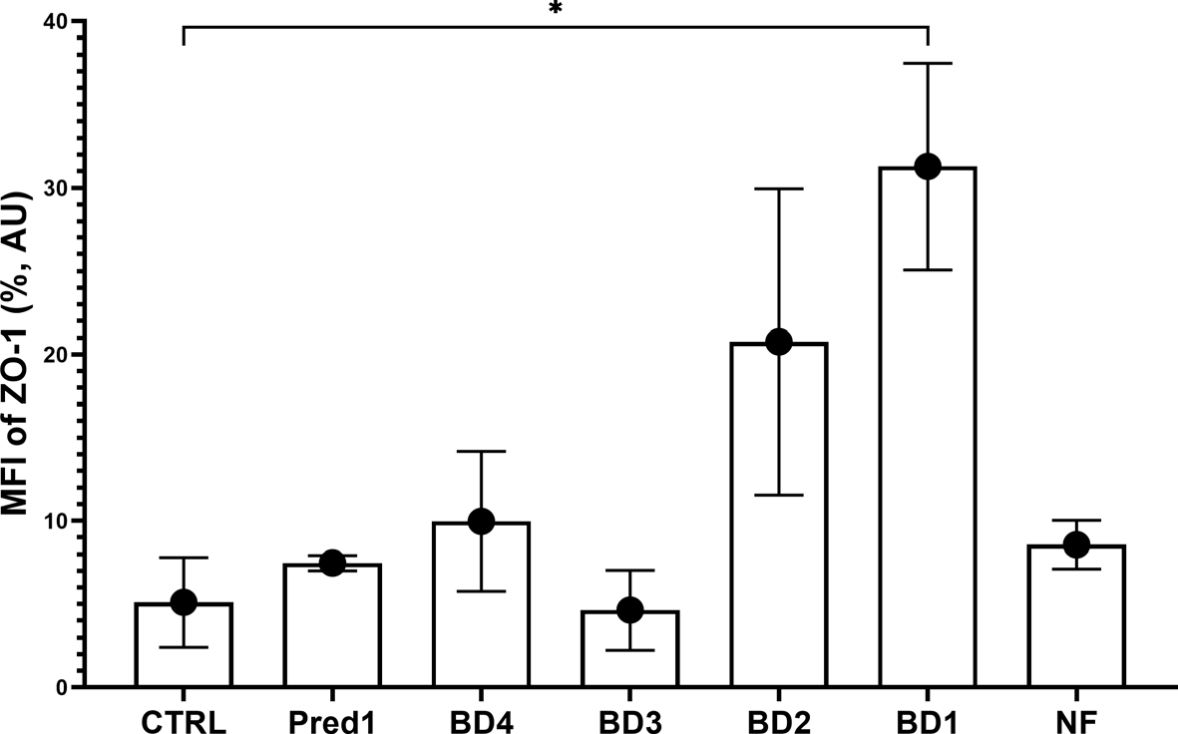
ZO-1 expression on the intestinal barrier. Graph shows the median fluorescence intensity of ZO-1 protein on the intestinal epithelial cells from each experimental group, compared to the control group. MFI, median fluorescence intensity; AU, arbitrary units; CTRL, control group; Pred1, prednisolone 1 µM; BD, broccoli digesta; NF, digesta no-food. Data are represented as Mean ± SD; n=3 independent experiments; *p<0.05 with respect to the CTRL group (Welch’s ANOVA tests, Dunnett’s T3 multiple comparisons test).

ZO-1 protein from the tight junctions was significantly increased on the intestinal epithelial cells by treatment with BD at the lowest concentration (BD1) compared to the control group, with no differences regarding the NF or 1 µM prednisolone treatments and a slight increase for BD4 and BD2 groups (**Figure 9**).

## 4 Discussion

In this study we developed and tested a tri-culture *in vitro* model of mild-inflammatory status, that included intestinal and immune cells, and is aimed to study the pro-/anti-inflammatory effects of *in vitro* digested foods. To that, three different cell lines have been contemporary assembled to simulate the physical and biochemical interactions naturally occurring *in vivo* between enterocyte-like cells that constitute the intestinal barrier, mucus-producing goblet-like cells and immune cells that trigger the inflammatory response. Over the past years, the most common gut cell model mimicking the intestinal barrier have been characterized by the use of single intestinal epithelial cell lines as Caco-2, mostly for particles toxicity analysis (20), drugs absorption and metabolism studies (34). The system has been improved over the years with the inclusion of an immune cell line, commonly human or mice monocytes or macrophages cell lines, allowing to simulate or evaluate several inflammatory processes (19, 21, 22). The 3C strategy, even if technically challenging, allows to overcome the simplistic issues derived from the single-cell or two-cells approaches (35). Moreover, it includes a z-axis, enhancing performance and showing cost-benefits and overall efficiency similar to 3D models, supporting the use of this model based on specific experimental requirements (36, 37).

As a first step, the intestinal epithelial barrier has been stablished by co-culturing the Caco-2 and HT29-MTX-E12 cells on Transwell inserts for up to 21 days, and assessed every 7 days (7, 14, 21 days) prior to the 3C assembly. Even if Caco-2 monolayers are a simple, cost-effective and rapid, reproducible tool to replicate the intestinal epithelial barrier, their use alone may cause an underestimation of some absorption processes, probably due to the lack of variety in the cell population with important roles (38). For instance, goblet cells – here represented by the HT29-MTX-E12 cell line – are the second most abundant population of cells in the intestinal epithelium, responsible for the mucus secretion that forms a layer covering the mucosal surface and acting as a physical barrier (39). Although Caco-2 and the mucus-producing goblet-like cells HT29-MTX-E12 co-culture should overcome some of the absorption problems associated, the main challenge described is the leak, since the co-culture may exhibit lower TEER values when increasing the HT29 cells proportion (40). To overcome this issue, in this study a 9:1 ratio (Caco-2: HT29-MTX-E12) has been used, simulating the proximal part of the intestine and ensuring a proper barrier permeability and mucus production, as supported by several researchers (41–46). Calculated TEER data were consistent with previously published data (40, 47), demonstrating that cells were correctly differentiated and the barrier was stable and intact.

To select the best inflammatory baseline conditions, the induction of a mild-inflammatory status by using multiple stressors at different concentrations has been attempted. A mild-inflammation has been preferred, instead of a high-inflammatory status, to induce a cytokine response able to be potentially modulated with the subsequent addition of food digesta thanks to their bioactive compounds. A first screening performed directly on THP-1 cells pointed out that 100 nM PMA stands out as the best option to induce a mild inflammatory status, stimulating THP-1 cells versus a macrophage phenotype, enabling their attachment to the surface and their shape modification, but without exceeding in the immune response in terms of TNF-α and IL-6 production. Then, the whole 3C model was assembled and stimulated both with LPS and PMA, at the concentrations previously tested. Supported by previous researchers (19, 48, 49), PMA addition to the basolateral compartment of the model has been selected as the best strategy to stimulate the immune cells and, in this specific environment, the whole intestinal model. PMA has been used at the concentration of 100 nM for 20 hours, inducing the release of a fair concentration of cytokines but reaching values in concordance with a mild-inflamed intestine, without reaching values that correlate with IBDs (50, 51), that may be reverted with a proper anti-inflammatory drug or food digesta. In fact, prednisolone has been used as an anti-inflammatory drug, frequently used for the treatment of IBDs such as Crohn disease thanks to its potential to normalize the intestinal permeability (52, 53). A glucocorticoid as prednisolone has been preferred instead of a non-steroidal anti-inflammatory drug due to their well-ascertained harmful effects on intestinal and gastric epithelial integrity (54, 55).

The extent and rate of nutrients absorption within the gastrointestinal tract, mainly by intestinal epithelial cells, depends on the digestion of the macronutrients and the generated products, with a considerable impact for the human physiological health (56). A key aspect of this study is the testing of food digesta instead of classical food extracts, commonly used *in vitro* for food research. Indeed, a great number of research articles in this field is based on the study of a single component extracted (using water or organic solvents) or diluted in a standard solution (usually a commercial buffer) or even lyophilized, disregarding the rest of the potential components in real food. For this reason, static and dynamic INFOGEST digestion models (32) have acquired an increased importance to study the gastrointestinal events related to the assumption of several types of foods and beverages, including fish, meat, vegetables, cereals, dairy, and other protein and lipid sources commonly consumed. Even though the complex dynamic processes naturally occurring *in vivo* and the digestion variations among the population groups (infants, adults, elderlies) are not exactly reproducible *in vitro*, this method represents a relatively simple, inexpensive and rapid tool to produce a digesta fluid mimicking the real digestive sample facing the apical membrane of the gut bowel. As a first analysis, the potential effects of the digesta solution with no food but containing all the enzymes needed for the digesta process was tested on the model, finding no differences in terms of TNF-α and IL-6 production.

To test the 3C model response to foods, and in particular the pro-/anti-inflammatory potential of foods, a BD was selected. Broccoli has been selected due to the benefits associated with its consumption. Several clinical trials have been conducted to evaluate its effects on human and other animals’ health (57–59), using either broccoli sprouts, powder supplementations or seed extracts (60, 61). Broccoli belongs to the cruciferous vegetables (CVs) of the Brassicaceae family, which have been widely studied for their anti-tumoral properties. Broccoli are a good source of vitamins, as C, E and K, as well as of bioactive compounds, including, as soluble and insoluble fibers, quercetin and kaempferol glycosides of flavonoids and a high content of glucosinolates, as glucoraphanin (4-methylsulphinylbutyl glucosinolate) and glucobrassicin (3-indolylmethyl glucosinolate). Among the hydrolysis byproducts known as isothiocyanates (ITC) (62–64) sulforaphane (SFN, 1-isothiocyanato-4-methylsulfinylbutane) stands out as the bioactive responsible for the anti-tumorigenic and anti-oxidant properties attributed to the CVs (60). SFN is a bioactive food component notably abundant especially in young broccoli sprouts, able to cause cell cycle arrest and apoptosis of cancer cells (65). Despite some limitations in its formation due to myrosinase enzyme activity as well as gut microbiota metabolism, *in vivo* studies have demonstrated how SFN reduces inflammatory markers and attenuate lipid peroxidation and oxidative stress in patients suffering from diabetes, improving fasting blood glucose levels and stabilizing insulin response (60, 66). *In vitro*, SFN inactivates the nuclear factor κβ (NF-kB) (67), which in turn downregulates the expression of pro-inflammatory cytokines production (68), attenuating the inflammatory response. Moreover, a recent study has shown that sulforaphane is able to change the growth of bacteria found in the gastrointestinal microbiota, altering some metabolites and producing anti-inflammatory molecules (69).

BD effects on the 3C have been evaluated in terms of cytotoxicity, intestinal barrier permeability and cytokine production. Cytokine analysis after BD treatments has confirmed the potential anti-inflammatory effects attributed to broccoli sprouts by several researchers (67, 68). In detail, BD strongly reduced the production of TNF-α, IL-6 and IL-8 from THP-1 cells after 4 hours of digesta treatment to the apical compartment of the system, in comparison with the control group and obtaining similar results to prednisolone. A reduction of pro-inflammatory cytokines has been described previously by other Authors, finding a downregulation of the release of TNF-α and IL-6 from LPS-stimulated human peripheral blood mononuclear cells (70) after treatments with extracts from broccoli sprouts, while Guo et al. found a reduction in IL-8 concentration after the treatment with an aqueous extract from broccoli seed in patients suffering from atrophic gastritis (71). Similar effects were described by Bessler and Djaldetti, attributing to SFN the ability to exert a concentration-dependent inhibitory effect on pro-inflammatory cytokines as TNF-α and IL-6 by PMBCs co-cultured with colon carcinoma cells (72). Regarding IL-18, a decreased concentration was found when treating with BD at the lowest concentration. IL-18 is released by monocytes to enhance intestinal inflammation upon NLRP3 inflammasome activation by Toll-like receptor 2 (TLR2), activated in turn by high-fat diets (73). IL-18 is implicated in several autoimmune diseases, as intestinal bowel diseases; however, its role in health and diseases is still not clear, with a growing number of studies supporting a protective role for IL-18 (74).

Through the last decades, individual factors as sex, age, body mass index (BMI), physical activity, smoke and certain dietary habits have been associated with the increase or decrease of specific cytokines. Although with controversial results (75), dietary patterns with high intakes of red meats, fried foods or processed ones have been generally associated with an increase of pro-inflammatory cytokines as TNF-α, IL-6 and IL-8, while diets rich in fruits and vegetables, with a high content of micronutrients, fiber and other bioactive components, i.e. polyphenols and glucosinolates, have been mostly associated with a decrease of pro-inflammatory cytokines and an increase of anti-inflammatory markers (76, 77).

As stated, BD treatments have been evaluated also in terms of cytotoxicity. LDH quantification assay has been performed to confirm the absence of toxicity for any of the concentrations used, using an indirect assay that measures, in a rapid and not expensive way, cell lysis without manipulating or damaging the cells (78). As observed, BD do not alter cells viability even at the higher concentrations.

Finally, to evaluate the barrier integrity and permeability after broccoli digesta treatments, the tight junction scaffolding protein zonula occludens-1 (ZO-1) was analyzed by flow cytometry by recovering the intestinal epithelial cells from the inserts after the experiments on the whole 3C model. ZO-1 is a member of the tight junctions system responsible for the cross-linking of its transmembrane proteins (as claudin and occludin) with the actin cytoskeleton (79). ZO-1 expression is downregulated in human and experimental inflammatory bowel disease, compromising mucosal repair and thus promoting disease progression (80). The results obtained in this study show an increase in ZO-1 protein in cells treated with the lowest BD concentration (BD1), highlighting the importance of the dose to achieve a desirable effect and the need for cellular markers to stablish an objective pro-/anti-inflammatory role for food digesta.

## 5 Conclusions

A reliable and promising 3C model to evaluate the pro-anti/inflammatory properties of digested foods after a process of *in vitro* digestion, mimicking a mild-inflammatory status, has been developed. Broccoli digesta was shown to modulate the release of pro-inflammatory cytokines and the tight junctions of the intestinal barrier by increasing the expression of the protein ZO-1. Although further digested foods should be tested and several additional cytokines may be investigated, the 3C model might be utilized for screening a wide array of food digesta to characterize the pro/anti-inflammatory effect of single foods, contributing to unravel the role of diet in modulating chronic inflammation.

## Data Availability

The raw data supporting the conclusions of this article will be made available by the authors, without undue reservation.
